# Demographic, anthropometric and intrasubject variations affect platelet‐rich plasma formulation

**DOI:** 10.1002/jeo2.70024

**Published:** 2025-01-26

**Authors:** Luis García‐Bordes, Pedro Álvarez‐Díaz, Eduard Alentorn‐Geli, Alfred Ferré‐Aniorte, Patricia Laiz‐Boada, Roberto Seijas‐Vázquez, Ramon Cugat‐Bertomeu

**Affiliations:** ^1^ Instituto Cugat, Hospital Quironsalud Barcelona Barcelona Spain; ^2^ Fundación García Cugat Barcelona Spain; ^3^ Universitat Internacional de Catalunya, Sant Cugat del Vallès Barcelona Spain; ^4^ Mutualidad de Futbolistas – Delegación Cataluña, Federación Española de Fútbol Barcelona Spain

**Keywords:** age, body mass index, growth factors, platelet concentration, platelet‐rich plasma, sex

## Abstract

**Purpose:**

The purpose of this study is to describe the inter‐ and intra‐individual differences in the platelet concentration between blood and platelet‐rich plasma (PRP) preparation, assess intersubject differences considering demographic and anthropometric variables, describe PRP code distribution and analyse intrasubject variability.

**Methods:**

A retrospective analysis was conducted using a single‐centre patient database from November 2021 to November 2023. It included patients with musculoskeletal pathologies treated with PRP injections. Primary variables were demographic characteristics (sex, age, body mass index [BMI]) and platelet concentrations in blood and PRP during treatments. Secondary analysis focused on PRP code frequency distribution and intrasubject variability according to different coding systems.

**Results:**

Here, 686 patients met the inclusion criteria. PRP exhibited significantly higher platelet concentrations compared to blood (378.01 ± 136.25 × 10^3^ platelets/µL vs. 221.97 ± 58.21 × 10^3^ platelets/µL, *p* < 0.001). Younger patients had higher platelet concentrations in both blood (*p* = 0.004) and PRP (*p* = 0.003), whereas female patients showed higher platelet concentrations only in blood (*p* < 0.001). The platelet concentration ratio was higher in males (*p* < 0.001) and those with higher BMI (*p* = 0.023). Significant differences were found between the existing and modified PRP coding systems (*p* < 0.001). Intrasubject variability was higher in PRP than in blood (coefficient of variance: 21.32 ± 17.36 in blood vs. 27.85 ± 19.10 in PRP, *p* < 0.001).

**Conclusion:**

Age, gender, BMI and intrasubject variations significantly affect PRP formulation, emphasizing the importance of addressing these variables for a more predictable, personalized and effective therapeutic approach.

**Level of Evidence:**

This is a retrospective study. Level IV.

**Abbreviations**
BMI: body mass index
CV: coefficient of variation
PRP: platelet‐rich plasma
RBCs: red blood cells
WBCs: white blood cells

## INTRODUCTION

Platelet‐rich plasma (PRP) is a plasma fraction with an increased concentration of platelets obtained through the centrifugation of whole blood [37]. It is known for its straightforward preparation, good tolerability and low complication rates, making it a popular option in both human and veterinary medicine [16, 31]. PRP has been widely used in various medical specialties, including cardiology, dentistry and maxillofacial surgery [5, 14, 18, 38, 56]. In recent years, its application in orthopaedics has grown significantly, particularly in the field of regenerative medicine [30, 39]. PRP is rich in growth factors [7, 8] that are believed to promote regenerative and anti‐inflammatory effects, aiding tissue healing and pain reduction [19]. Studies have shown that PRP can improve outcomes in bone regeneration [25, 40], chondrocyte proliferation [2] and mesenchymal differentiation [54], among other processes [1, 48]. Clinical trials indicate that intra‐articular PRP injections can effectively reduce pain in the short to medium term, outperforming treatments like hyaluronic acid [21] and showing long‐term benefits for tendinopathies compared to corticosteroids [23]. However, the lack of standardization in PRP preparation, composition [24, 39, 47, 52] and administration [6, 31, 37, 38, 59] has led to variability in results and inconclusive evidence regarding its overall effectiveness [3, 9, 13, 21, 41, 46, 50, 53]. A PRP classification system has been proposed to address these issues and enhance consistency in clinical outcomes [27].

In view of the number of factors impacting PRP outcomes and the ambiguity surrounding its clinical efficacy, there is a need for robust studies to fully understand its complete clinical potential and the possible inter‐ and intrapatient variations. Understanding the roles of age, sex and body mass index (BMI) in PRP composition is critical to ensure more predictable and effective therapeutic outcomes [38, 47, 51, 52, 55].

Thus, the primary aim of this study is to describe and compare the platelet concentration between blood and PRP among patients receiving PRP treatment. It also aims to assess the intersubject differences through the influence of demographic and anthropometric variables on PRP characteristics, describe the distribution and frequency of PRP codes based on two coding systems, and analyse the intrasubject variability of platelet concentration across successive PRP treatments.

## MATERIALS AND METHODS

The study was designed as a retrospective analysis of a prospective, single‐centre, patient database cohort. The primary hypothesis of this study is that the platelet concentration in PRP is significantly higher than in whole blood and this concentration is influenced by demographic and anthropometric variables such as age, sex and BMI. Additionally, the study proposes several secondary hypotheses. First, it hypothesizes that the method of PRP coding—whether by rounding or truncation—significantly affects the distribution of PRP codes. Lastly, the study hypothesizes that intrasubject variability in platelet concentration is higher in PRP compared to whole blood. These hypotheses are designed to explore the variability and influencing factors in PRP preparation, aiming for more personalized and effective therapeutic approaches.

### Participants

Inclusion and exclusion criteria were established to ensure that study participants represented the population that would benefit from PRP treatment and to minimize risks and variables that could affect the study outcomes.

The inclusion criteria for this study were as follows: all consecutive subjects treated with PRP within the age range of 18–75 years for any osteoarticular pathology between November 2021 and November 2023, which had not improved with prior conservative treatments. All participants had to be capable of providing written informed consent and be in an overall good health condition suitable for undergoing treatment and follow‐up.

The exclusion criteria included the following: patients with severe or uncontrolled systemic diseases, such as cardiovascular diseases, uncontrolled diabetes mellitus, haematological disorders, oncological pathology, or active infections, were excluded. Those with coagulation disorders, thrombocytopenia or platelet dysfunction, which could affect the efficacy or safety of PRP, were also not eligible. Patients undergoing treatment with anticoagulants or antiplatelet agents were excluded, as well as those with local or systemic infections at the injection site. Patients with conditions requiring immediate surgery were not included. Furthermore, individuals who had received corticosteroid injections, hyaluronic acid or other intra‐articular treatments within a 3‐month period before the study were excluded. Women who were pregnant or breastfeeding were not eligible for the study. Lastly, any patients who were unable to comply with the study's follow‐up protocol were also excluded.

PRP treatment was prescribed during a routine medical consultation by the patient's physician. Depending on the pathology, it can involve a single PRP infiltration or a series of three consecutive PRP injections, with a 2‐week interval between each session. PRP counting is performed after every injection and registered at the data processing software. This study obtained approval from the Institutional Review Board.

### PRP preparation method

PRP preparation was performed using the Endoret^©^ PRGF^©^ system (BTI Biotechnology Institute). Before treatment, blood samples were obtained in 4 h fasting conditions and distributed into eight 9 mL tubes, each containing a 3.8% citrate solution. Subsequently, a BTI System IV^©^ centrifuge was used for 8 minutes at 580 g (single spin), resulting in the sedimentation of red and white blood cells (WBCs) at the bottom, whereas platelets along with plasma were concentrated at the top portion of the tubes.

During the extraction, an additional 9 mL tube with EDTA was collected, along with 1 mL of the final PRP, for sample counting purposes. A haematological counter (Shenzhen Dymind Biotechnology Co., Ltd) was employed for this counting. This device is a quantitative, automated haematology analyser designed for blood cell counting, three‐part classification of WBCs and measurement of haemoglobin concentration. This counter is associated with a data processing software (BioSmartData) that allows the classification of PRP according to Kon et al. [27]. PRP counting was performed after every infiltration and registered at the data processing software.

All PRP samples have been homogeneously collected, prepared, registered and administered by the same medical and nursing team, and under the same automatized protocol, minimizing the errors related to data registration.

### Variables

At the time of the first medical consultation and after PRP treatment prescription was prescribed, demographic and anthropometric variables were collected, including age, sex, BMI, the index musculoskeletal disorder and laterality. Platelet concentration was analysed in both the blood sample and the final PRP product for all patients (interindividual variability) after every PRP injection and displayed as 10^3^ platelets/µL. In case of more than one injection per patient, platelet concentration analysis was repeated accordingly. Intrasubject variability of platelet concentration across successive PRP treatments was calculated in patients with a minimum of three repeated injections, to determine whether platelet concentrations change over time in the same patient. In addition, in order to study the increment of platelet concentration between blood and PRP, the platelet concentration ratio was calculated by dividing the platelet concentration in PRP by that in blood.

The final PRP product was classified using the PRP coding system [27]. Briefly, such a code system consists of a six‐digit number containing information about platelet concentration in blood, platelet concentration in PRP, red blood cell (RBC) concentration in PRP, WBC concentration in PRP, activation and use of calcium chloride for activation. This classification system has to be calculated, according to the authors, by truncation method. That is, the digit for platelet concentration would be the same as the digit in the hundred position. As an example, in case of a platelet concentration of 285 × 10^3^ platelets/µL, the correspondent digit in the PRP code system would be ‘2’. In order to minimize this intrinsic margin of error during this calculation, a rounding instead of truncation method was proposed. Following the previous example, a ‘3’ digit would be given for the PRP coding system as per the rounding numbers method. The PRP code frequency of each method was compared to evaluate if significant differences can be found between both rounding and truncation methods.

### Statistical analysis

Descriptive statistics were used to summarize demographic, anthropometric and injury characteristics. Qualitative variables were expressed as numbers and percentages, while quantitative variables were expressed as mean ± SD.

The Shapiro–Wilk test was used to evaluate the normal distribution of variables. For normally distributed quantitative variables, an unpaired *t* test was used to evaluate between‐group differences. In the case of nonnormal distribution, a Wilcoxon test was employed. For the analysis of quantitative variables against quantitative variables, Pearson's correlation coefficient was used for variables with normal distributions, while Spearman correlation was used for variables with nonnormal distributions. If the correlation coefficient ranged between 0.9 and 1.0, it was considered very high; if ranged between 0.7 and 0.9, it was considered high; if ranged between 0.5 and 0.7, it was considered moderated; if ranged between 0.3 and 0.5, it was considered as low; and if ranged between 0.3 and 0, it was considered as very low. Finally, for the intrasubject variations in platelet concentration, the coefficient of variation (CV) was applied. *χ* ^2^ analysis was used to compare the distribution of the two proposed PRP coding systems. Only those PRP codes that accounted for more than 1.5% of the total were included in the analysis. The significance level was set at 0.05. All the statistical analyses were conducted with SPSS® Statistics software v.15 (SPSS Inc.).

## RESULTS

**Figure 1:**
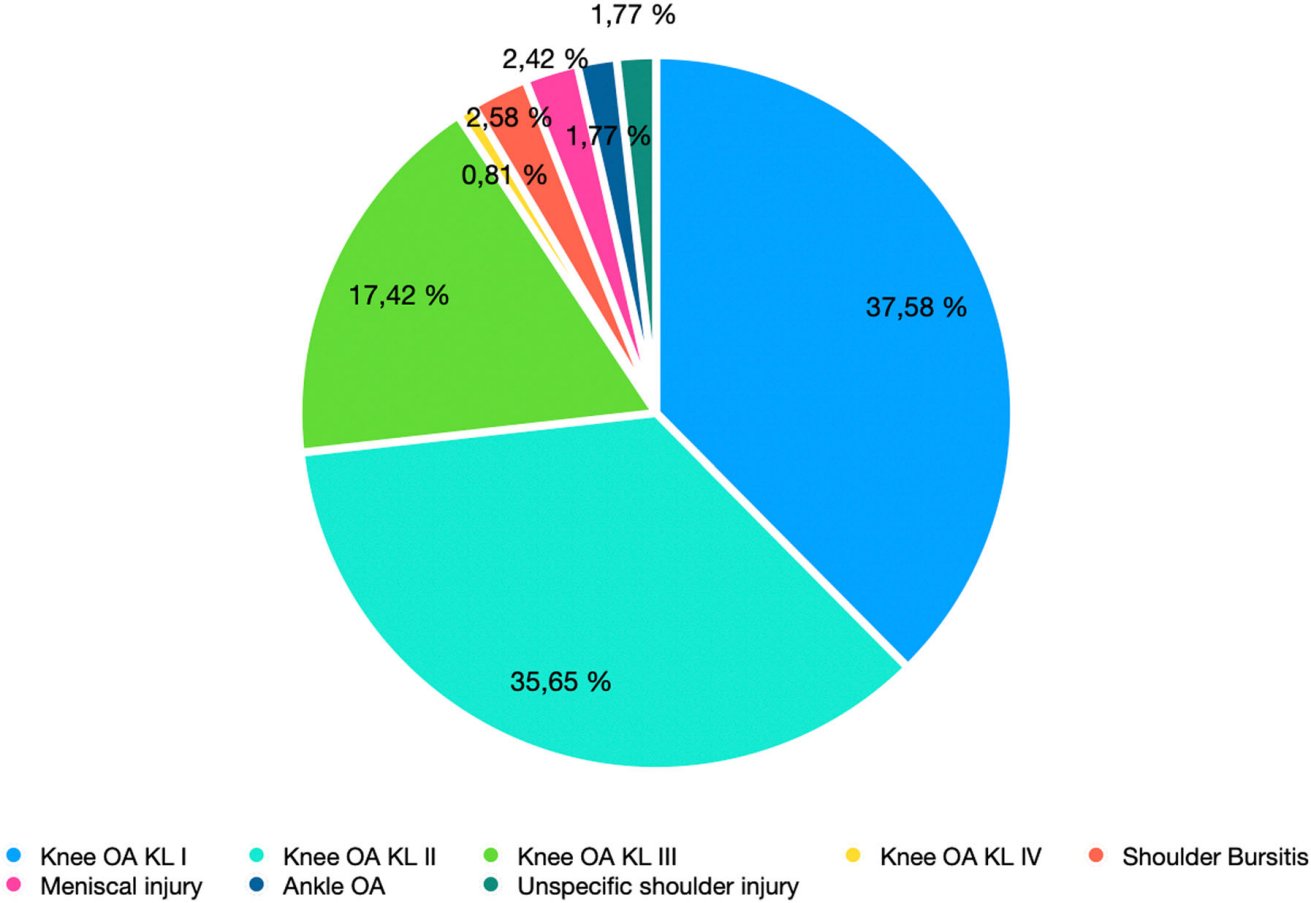
Pathologies included in the study. KL, Kellgren–Lawrence; OA, osteoarthritis.

A total of 686 patients were treated with PRP injections and were included in the analysis. Patient anthropometric, demographic and injury characteristics are shown in Table 1 and Figure 1.

**Table 1:** Anthropometric and demographic characteristics.

Age (mean ± SD), years	58.7 ± 15.4
BMI (mean ± SD)	26.7 ± 4.6
Sex	
Male, *N* (%)	333 (48.5%)
Female, *N* (%)	353 (51.5%)
Laterality	
Bilateral, *N* (%)	351 (51.2%)
Right, *N* (%)	188 (27.4%)
Left, *N* (%)	143 (20.8%)

Abbreviation: BMI, body mass index.

A significantly higher platelet concentration was found in PRP (378.0 ± 136.2 × 10^3^ platelets/µL) compared to blood (221.9 ± 58.2 × 10^3^ platelets/µL) (*p* < 0.001).

**Figure 2:**
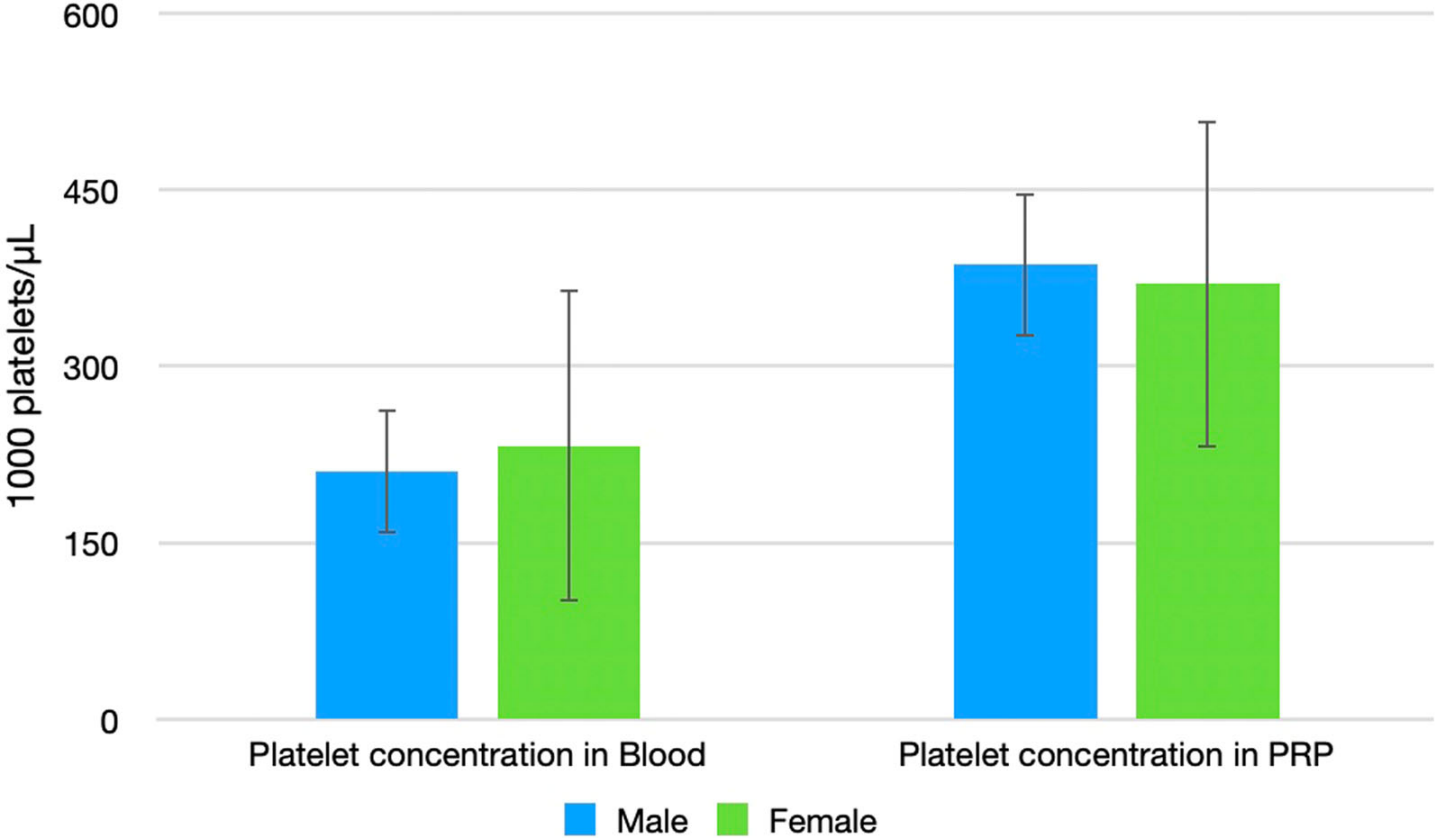
Platelet concentration in blood and PRP by sex. Female patients show significantly higher platelet concentration in blood than male patients. PRP, platelet‐rich plasma; 10^3^ platelets/µL.

The analysis revealed a statistically significant negative but very low correlation between age and platelet concentration in both blood and PRP, meaning younger patients manifested increased platelet concentrations in both blood and PRP, and female patients showed significantly higher platelet concentration in blood than male patients (Figure 2). No significant correlations were found between BMI and platelet concentrations in either blood (*p* = 0.450) or PRP (*p* = 0.213) (Table 2). Homogeneity analysis revealed significantly younger ages in the male group but no differences for BMI between males and females (Table 3).

**Table 2:** Relationship between demographic and anthropometric variables and platelet concentration in blood and PRP.

Variable	Platelet concentration in blood	*p*	Platelet concentration in PRP	*p*
Age	−0.114^a^	0.004	−0.119^a^	0.003
BMI	−0.033^a^	0.450	0.054^a^	0.213
Sex				
Male (mean ± SD)^b^	210.8 ± 53.0	<0.001	386.3 ± 132.7	0.119
Female (mean ± SD)^a^	232.5 ± 60.9		370.1 ± 139.1	

*Note*: The analysis reveals a statistically significant negative but very low correlation between age and platelet concentration in both blood and PRP, meaning younger patients manifested increased platelet concentrations in both blood and PRP. No significant correlations were found between BMI and platelet concentrations in either blood or PRP.

Abbreviations: BMI, body mass index; PRP, platelet‐rich plasma.

^a^ Pearson's correlation coefficient.

^b^ 10^3^ platelets/µL.

**Table 3:** Homogeneity analysis reveals significantly younger ages in the male group but no differences for BMI between males and females.

	Male	Female	*p*
Age (mean ± SD)^a^	55.9 ± 15.2	61.4 ± 15.2	<0.001
BMI (mean ± SD)^a^	26.9 ± 4.1	26.4 ± 5.0	0.029

Abbreviation: BMI, body mass index.

^a^ 10^3^ platelets/µL.

The platelet concentration ratio was 1.80 ± 0.93, with male patients showing a statistically significant increase compared to female patients. A significant positive correlation was found between BMI and platelet concentration ratio (*p* = 0.023) (Table 4).

**Table 4:** Relationship between demographic and anthropometric variables and the platelet concentration ratio.

Variable	Platelet concentration ratio	*p*
Age	0.004^a^	0.918
BMI	0.098^a^	0.023
Sex		
Male (mean ± SD)	1.93 ± 1.00	<0.001
Female (mean ± SD)	1.68 ± 0.83	

*Note*: Male patients show a statistically significant increase compared to female patients. A significant positive correlation was found between BMI and platelet concentration ratio.

Abbreviation: BMI, body mass index.

^a^ Pearson's correlation coefficient.

A total amount of 260 patients from the initial 686 patients performed a minimum of three consecutive PRP infiltrations and were included in the intrasubject analysis of the variability of platelet concentration. The CV was significantly lower in blood compared to PRP, with values of 21.32 ± 17.36 and 27.85 ± 19.10, respectively (*p* < 0.001) (Table 5).

**Table 5:** CV of platelet concentrations in patients with successive PRP treatments.

Blood		PRP		
Mean ± SD^a^	CV	Mean ± SD^a^	CV	*p*
214.8 ± 37.5	21.3 ± 17.3	373.2 ± 91.2	27.8 ± 19.1	<0.001

Abbreviations: CV, coefficient of variation; PRP, platelet‐rich plasma.

^a^ 10^3^ platelets/µL.

The results of the PRP coding system analysis showed that the most common PRP codes were those doubling platelet concentration in PRP compared to blood, including 12‐00‐11, 24‐00‐11 and 36‐00‐11. None of the codes showed presence of either WBC or RBC. Additionally, all codes reflected the activation method with calcium chloride.

**Figure 3:**
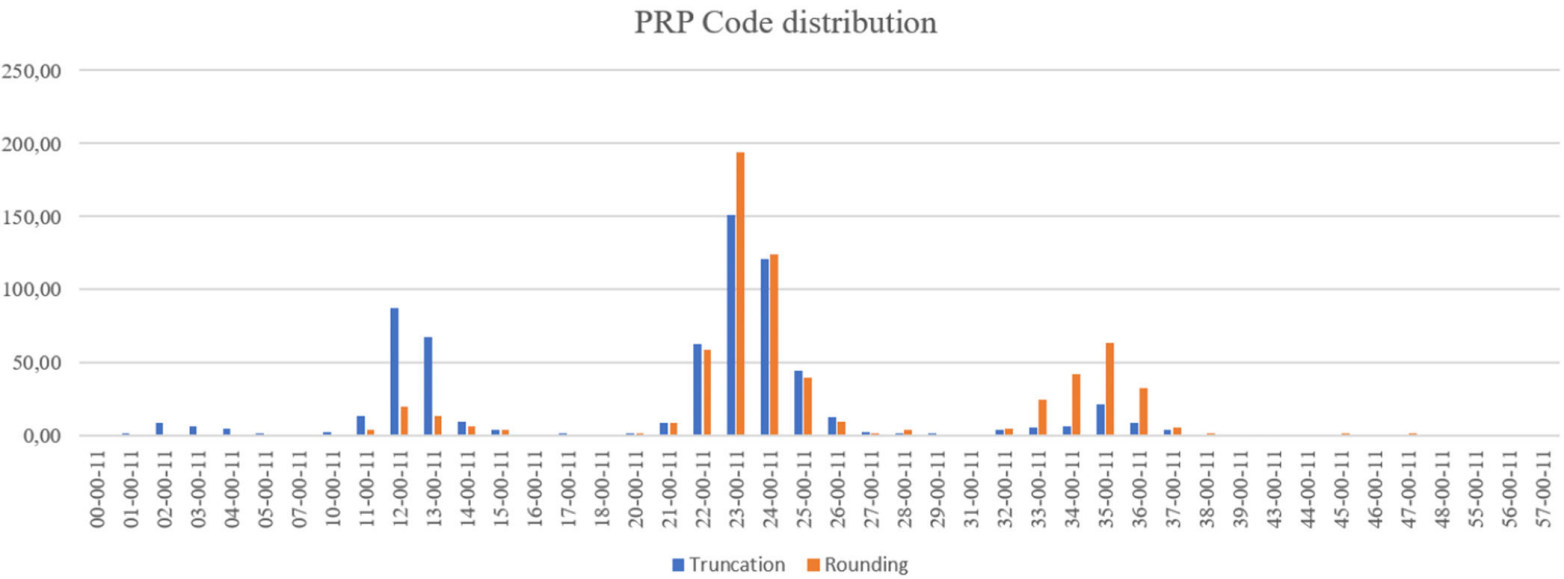
PRP code distribution using rounding and truncation methods. PRP, platelet‐rich plasma.

Statistically significant differences were observed when comparing the distribution of the two proposed PRP coding systems (rounding and truncation methods); the truncation method over‐represented lower PRP codes above higher ones (*p* < 0.001) (Figure 3).

## DISCUSSION

The main finding of this study is that platelet concentration is not only higher in PRP compared to blood but is also influenced by patient's demographic and anthropometric characteristics. A significant negative correlation between age and platelet concentration in both blood and PRP is observed. Younger patients show higher platelet concentrations in blood and PRP (*p* < 0.05). Regarding sex, female patients demonstrate significantly higher platelet concentration in blood, acknowledging that homogeneity analysis reveal significantly younger ages in the male group. No significant differences have been found in platelet concentrations related to BMI. However, higher BMI and male sex are related to higher platelet concentration ratio.

The clinical efficacy of PRP therapy remains controversial due to the variability in PRP characteristics. The two primary factors considered in the literature that may come into play regarding these variations are the devices and the protocols used for PRP preparation. A multitude of commercial devices are currently available for the preparation of PRP [23, 47, 52]. Although their common objective is to obtain a final product with a higher platelet concentration than the whole blood, each device operates differently. This variability in the platelet concentration ratio, absence or presence of RBC and WBC, absence or presence of activation, and activation method are determined by whether it involves a one‐step or two‐step centrifugation, the type and operation of the collecting tube, the speed of the centrifuge and other production processes [11, 12, 25, 27, 44]. The preparation method of PRP used in this study involves a one‐step centrifugation process at 580*g* and activation with calcium chloride. Consequently, these variations result in PRP preparations with diverse volumes, platelet counts and concentrations of residual white and RBCs, contributing to the multifaceted landscape of PRPs [4, 11, 18, 33]. In addition, different application protocols in terms of number of injections, time between injections, long‐lasting placebo effects of injectables, concomitant treatment with anti‐inflammatory drugs, or specific location of the injection for a similar disorder may explain differences in clinical outcomes.

However, it is imperative to acknowledge that devices and protocols are not the sole contributing factors to the broad spectrum of biological activities associated with PRP. The presence or absence of mononuclear cells, neutrophils and RBCs remains the subject of debate and underscores the complexity of the topic. While some studies have found no significant influence of leucocytes in PRP when they are present [15, 49], other studies suggest that the presence of leucocytes may have beneficial antimicrobial effects or, conversely, adverse pro‐inflammatory effects depending on the clinical context [10, 60].

To address the need for a comprehensive evaluation of PRP and the standardization of its preparation, a PRP classification system has been introduced, aiming to appraise the composition of the final PRP product [27]. This classification categorizes PRP based on the injected platelet dose, production efficiency, purity and the activation method employed [20, 26, 34]. The results in this study show that the most frequent codes are those around doubling platelet concentrations in PRP compared to blood without RBC and WBC in the final PRP product (12‐00‐11, 24‐00‐11 and 36‐00‐11). Although useful and interesting, the PRP coding system developed by Kon et al. [27] uses a truncation method during its calculation, which implies an intrinsic higher margin of error than the rounding method. Thus, this study analyses the differences in the frequency distributions of PRP codes delivered by both methods (truncation and rounding). These results showed statistical differences between both, with the truncation method over‐representing lower PRP codes compared to the rounding method. Accordingly, it is recommended that practitioners and researchers in the field of PRP revise the PRP coding system according to the guidelines outlined in the current analysis.

Despite PRP typically being characterized as a plasma enriched with platelets at a concentration five times greater than that found in whole blood [35, 36, 52], the precise PRP concentration required for optimal effectiveness remains enigmatic, ranging from greater than 200 to 1000 × 10^3^ cells/µL [36, 38] (which is approximately two to six times the baseline concentration in normal blood)**.** The most efficacious platelet concentration for tissue healing is typically found to be in the range of 200,000–1,000,000 platelets/µL [37]. This concentration has been shown to optimize the release of growth factors and cytokines. A higher concentration or absolute number of platelets within PRP does not necessarily lead to an enhanced tissue healing effect, since the dose–response curve is not linear, and a saturation effect has been described in which an inhibitory cascade ensues once a sufficiently high concentration of platelets is reached [39]. In fact, Giusti et al. [22] proposed that the most efficacious platelet concentration for the stimulation of tissue angiogenesis is 1.5 × 10^6^ platelets/µL. Theoretical levels of platelet‐derived growth factors in PRP might be expected to depend on the number of platelets involved. However, does not show a statistically significant correlation between platelet count and growth factor levels [55]. This result might be explained by high individual variability in cellular production or storage of cytokines [55]. On the other hand, from a clinical‐response point of view, recent studies argue the opposite for osteoarthritis, in which higher platelet concentrations achieve better clinical outcomes [42, 43].

The influence of PRP quality on clinical outcomes presents a complex landscape characterized by intrasubject and intersubject variability. Variations between subjects can be related to different factors such as age or sex [17, 29, 33, 39, 47, 51, 52, 55, 57, 58]. The complexity of this sex‐based variations significantly influences the multifaceted effects of PRP [28, 32]. Our results elucidate significant differences in the ability to increase platelet concentration in PRP compared to blood based on sex and BMI. Men have a significantly higher platelet concentration ratio than women (1.93 vs. 1.68, respectively; *p* < 0.001). On the other hand, there is a significantly positive correlation between BMI and the platelet concentration ratio, meaning that patients with a higher BMI can concentrate more platelets in PRP compared to blood (correlation = 0.098, *p* = 0.023), unlike what is reported in other studies [47].

Both blood and PRP quality varies also between different samples on the same patient. The CV is quite high in both cases: 21.32% in blood and 27.85% in PRP, similar to those observed in the literature [47]. The difference between both is significant, so we can conclude that the variation of platelets in PRP is higher than the variation of platelets in blood.

This study has some limitations. Since age, BMI and sex influence PRP platelet concentration and platelet concentration ratio, a homogeneity analysis was performed to examine whether male and female groups differ in BMI or age, which could affect the results. It was observed that males presented a significantly younger age (~5.5 years) compared to females. Regarding BMI, males presented a significantly higher BMI than females, with a difference of ~0.45. Although statistically significant, those differences could not be large enough to influence in a clinical setting. However, further confirmatory studies should be performed in homogeneous samples. Another potential confounding effect is that the timing of the PRP treatment was not recorded [19, 45]. Finally, there has been no control over possible systemic conditions that could have affected the platelet concentration in both blood and PRP. Further studies should take into account the presence or absence of such medical conditions in the final PRP characteristics. This study has also several strengths. The sample size is large enough to sustain the conclusions obtained when compared to previous studies. The data included in the analysis has also been obtained in a homogeneous way, by the same medical and nursing team and under the same automatized protocol, minimizing the errors related to data registration.

## CONCLUSION

The results presented in this study suggest that age, gender, BMI and intrasubject variations significantly affect the PRP formulation, emphasizing the importance of addressing these variables to achieve a more predictable and effective therapeutic approach. These findings contribute to a better understanding of the individualization of PRP treatment, paving the way for more informed and personalized treatment strategies in musculoskeletal regenerative medicine.

## AUTHOR CONTRIBUTIONS

**Luis García‐Bordes**: Conceptualization; methodology; writing—original draft; writing—review and editing; final manuscript revision. **Pedro Álvarez‐Díaz**: Review; supervision; final manuscript revision. **Eduard Alentorn‐Geli**: Writing—review and editing; final manuscript revision. **Alfred Ferré‐Aniorte**: Writing—review; data curation; formal analysis; final manuscript revision. **Patricia Laiz‐Boada**: Writing—review; data curation; final manuscript revision. **Roberto Seijas‐Vázquez**: Review; supervision; final manuscript revision. **Ramón Cugat‐Bertomeu**: Supervision; final manuscript revision.

## CONFLICT OF INTEREST STATEMENT

The authors declare no conflict of interest.

## ETHICS STATEMENT

This study was approved by the COMITÉ ÉTICO DE INVESTIGACIÓN (CEIm) GRUPO HOSPITALARIO QUIRÓNSALUD‐CATALUNYA, approval number SET‐PRP‐2021‐01.

## Data Availability

All data generated or analysed during this study will be available upon request.
